# *In vitro* activity of MRS-2541, a novel MetRS inhibitor, against a selection of resistant gram-positive organisms associated with serious hospital infections

**DOI:** 10.1128/spectrum.03615-25

**Published:** 2026-03-11

**Authors:** John Domagala, Seyedhameneh Jahanbakhsh, Angela K. Mendez, Chris M. Pillar, Andrea Marra, David A. Hufnagel, Nora M. R. Molasky, Zhongsheng Zhang, Erkang Fan, Dawn Reyna, Elke Lipka, Frederick S. Buckner

**Affiliations:** 1 TSRL Inc., Ann Arbor, Michigan, USA; 2 Microbiologics, Inc., Bacteriology and Mycology Center of Excellence, Portage, Michigan, USA; 3 CERID, Division of Allergy and Infectious Diseases, University of Washington 7284, Seattle, Washington, USA; 4 Department of Biochemistry, University of Washington 7284, Seattle, Washington, USA; Sacramento County Public Health Laboratory, Sacramento, California, USA

**Keywords:** antibiotics, gram-positive, *Staphylococcus*, *Streptococcus*, *Enterococcus*, tRNA synthetase, MetRS inhibitors

## Abstract

New antibacterials are still needed to treat serious gram-positive hospital infections, including acute bacterial skin and skin structure infections and bloodstream infections. MRS-2541 is a novel type 1 methionyl-tRNA synthetase inhibitor highly selective for gram-positive bacteria. MRS-2541 was evaluated with multiple comparator antibiotics against a large panel of gram-positive organisms enriched with antibacterial-resistant phenotypes. MRS-2541 was the most potent agent against 45 *Staphylococcus aureus* (SA) (6 SAs, 27 methicillin-resistant *Staphylococcus aureus*, and 12 vancomycin-intermediate *S. aureus*) and 35 coagulase-negative staphylococci with MIC_90_ values of 0.06 and 0.12 µg/mL, respectively, which was superior to all comparators, including vancomycin, linezolid, and daptomycin. MRS-2541 was very potent against 15 *Enterococcus faecalis* (4-VRE) and 13/15 *Enterococcus faecium* at ≤0.016 µg/mL, with two insensitive *faecium* strains (>16 µg/mL). MRS-2541 was less potent against *Streptococcus* spp., with MIC_90_ values vs *S. pyogenes*, *S. pneumoniae*, and *S. sanguinis* of 0.5 μg/mL. *S. agalactiae* and the viridans group streptococci were much less sensitive, with MIC_90_ values of 8 to >16 µg/mL. These data support MRS-2541 as a new potential option for serious gram-positive hospital infections.

**IMPORTANCE:**

Infections caused by antibiotic-resistant bacteria are inherently challenging to treat. Poor outcomes, including higher death rates, are associated with infections caused by antibiotic-resistant organisms. As a result, there is an urgent need for new antibiotics, particularly ones that act via novel mechanisms that evade established resistance mechanisms. In this paper, we provide new data about MRS-2541, a preclinical antibiotic that acts by inhibiting the bacterial methionyl-tRNA synthetase. We show that MRS-2541 has potent in vitro activity against diverse strains of *Staphylococcus*, *Streptococcus*, and *Enterococcus*. For example, all 115 strains of *Staphylococcus* spp. (including 27 methicillin-resistant *Staphylococcus aureus* strains and 12 vancomycin-intermediate *S. aureus* strains) were fully susceptible to MRS-2541 with MICs of <0.25 μg/mL. The specific activity against gram-positive bacteria and sparing of gram-negative flora indicates that MRS-2541 may be less disruptive of intestinal microbiota than broad-spectrum antibiotics. MRS-2541 is a promising preclinical candidate for infections due to resistant gram-positive organisms.

## INTRODUCTION

Globally, there are over 55 million acute bacterial skin and skin structure infections (ABSSSIs) per year (1). In the United States, ABSSSIs account for over 14 million healthcare visits per year, with 4 million emergency department visits and over 900,000 yearly hospital admissions (1, 2). The incidence continues to rise with the increasing aged population due to multiple risk factors such as diabetes, COPD, and prior exposure to healthcare facilities (2–4). Moreover, the incidence of recurrent infection is 26%, and the all-cause mortality rate in 2019 was 3.6% (5), which places an enormous burden on the healthcare system of over $14 billion (2). ABSSSIs are caused almost exclusively by staphylococci and streptococci, with *Staphylococcus aureus* (SA) and *Streptococcus pyogenes* being the most common pathogens (2, 6). Methicillin-resistant *Staphylococcus aureus* (MRSA) is much more difficult to treat and comprises 20% to 74% of the SA infections around the world (7). Of great concern is that 6% to 27% of hospital-treated skin infections lead to bloodstream infections (BSIs) and sepsis (5), MRSA infections having a 50% greater chance than methicillin-sensitive *S. aureus* (MSSA) infections for this outcome, with 15% to 60% mortality (8). Beyond ABSSSIs and BSIs, there are growing medical needs to treat other hospital infections caused by multidrug-resistant *S. pyogenes*, especially *Enterococcus faecalis* and *faecium*, where mortality is between 20% and 50% (9, 10).

While serious hospital infections are almost always treated with an IV drug immediately, switching to an oral step-down therapy after initial success with IV administration has major medical benefits and cost savings, and it is ideal when the switch is to the same agent (11, 12). The longstanding agent of choice for treating serious hospitalized gram-positive infections is vancomycin (no oral step down); however, rates of resistance or intermediate resistance to SA strains have risen to >5% and continue to climb (9, 13). While new antibiotics covering gram-positive infections have been introduced in the past decade, all but one are from established drug classes (fluoroquinolones, tetracyclines, oxazolidinones, and glycopeptides), and the risk of resistance remains significant for these classes (14, 15). As of 2021, there were 36 drugs approved for ABSSSIs (14, 15), with 32 new compounds in the pipeline, but only three of the pipeline compounds are small molecules with new mechanisms of action (16). Despite all the approved agents for ABSSSIs, its management remains challenging with inappropriate treatment in up to 25% of cases (17). Many of the treatments are only moderately effective, due in large part to the emergence of resistant bacteria and very few new compounds with novel mechanisms of action (2, 14, 17).

We recently reported a new, broad-spectrum gram-positive agent, MRS-2541, for oral and IV administration (18). MRS-2541 is a highly potent (sub-nanomolar) small molecule inhibitor of type 1 methionyl-tRNA synthetase, which is an essential enzyme conserved across gram-positive pathogens, including MRSA and multidrug-resistant enterococci. The type 2 methionyl-tRNA synthetase is present in gram-negative bacteria and the cytosol of eukaryotes. A few gram-positive species, like *S. pneumoniae*, have both enzymes (19). As expected, MRS-2541 is inactive vs gram-negative bacteria and less active vs organisms that contain both enzymes (18). The MRS enzymes attach methionine to the corresponding tRNA^met^ that is used at the ribosome for protein initiation and translation. Against an initial test panel, MRS-2541 displayed MICs versus *S. aureus* and *E. faecalis* of ≤0.125 µg/mL with a >100-fold selectivity index in mammalian cell cytotoxicity assays. It had good oral bioavailability with extensive tissue distribution in rodents and showed efficacy comparable to linezolid, with both oral and subcutaneous dosing in murine models (18). While the initial MICs vs laboratory strains indicated the potential for broad gram-positive activity, we desired to examine a larger panel of clinical isolates biased with resistant phenotypes, which, though rare in the general population, provide valuable information on the full spectrum of MRS-2541. Most of the previous MetRS inhibitor literature (18, 20, 21) accentuated the excellent *S. aureus* and MRSA activity, but for the first time, we also report on a full panel of staphylococci species as well as MDR streptococci and enterococci, given their growing role in gram-positive hospital infections (9, 10).

## RESULTS

The test article MRS-2541 was evaluated for *in vitro* activity alongside the appropriate comparators against large panels of gram-positive isolates, including those with defined resistance phenotypes. The test panels were enriched for antimicrobial resistance (AMR) phenotypes (i.e., MRSA, vancomycin-intermediate *S. aureus* [VISA], vancomycin-resistant *Enterococcus* [VRE], and penicillin-resistant *S. pneumoniae* [PRSP]) that can be seen in the line listings, along with individual MIC values and quality control (QC) testing (Tables S1 to S3). The results of testing MRS-2541 can be found in Tables 1 and 2 (staphylococci), Table 3 (enterococci), and Tables 4 and 5 (streptococci).

**TABLE 1:** Summary of the activity of MRS-2541 and comparators against *Staphylococcus* spp. isolates^*a*^

Organism	*N*	Drug	Phenotype	MIC (µg/mL)			S/I/R (%)
				Range	MIC_50_	MIC_90_	
*S. aureus*	45	MRS-2541	Overall	≤0.016 to 0.12	0.03	0.06	–
			MSSA (*N* = 18)	≤0.016 to 0.12	0.03	0.06	–
			MRSA (*N* = 27)	≤0.016 to 0.06	0.03	0.06	–
			VISA/hVISA (*N* = 12)	≤0.016 to 0.06	0.03	0.06	–
		Levofloxacin	Overall	0.03 to >16	2	>16	44.4/4.4/51.1
		Oxacillin	Overall	0.12 to >32	16	>32	40.0/–/60.0
		Erythromycin	Overall	0.25 to >16	>16	>16	33.3/0/66.7
		Clindamycin	Overall	0.06 to >16	0.12	>16	66.7/0/33.3
		Vancomycin	Overall	0.25 to 8	0.5	4	77.8/22.2/0.0
		Daptomycin	Overall	0.5 to 4	1	2	86.7/–/–
		Linezolid	Overall	1 to 4	2	2	100/–/0.0
CoNS	35	MRS-2541	Overall	0.06 to 0.25	0.12	0.12	–
			MSCoNS (*N* = 14)	0.06 to 0.12	0.12	0.12	–
			MRCoNS (*N* = 21)	0.06 to 0.25	0.12	0.12	**–**
		Levofloxacin	Overall	0.06 to >16	2	16	48.6/5.7/45.7
		Oxacillin	Overall	0.06 to >32	4	>32	40.0/–/60.0
		Erythromycin	Overall	0.12 to >16	>16	>16	2.9/22.8/74.3
		Clindamycin	Overall	0.06 to >16	0.12	>16	62.9/2.9/34.3
		Vancomycin	Overall	0.5 to 2	1	2	100/0.0/0.0
		Daptomycin	Overall	0.25 to 1	0.5	1	100/–/–
		Linezolid	Overall	0.5 to 2	1	1	100/–/0.0

^*a*^ CoNS, coagulase-negative *S. aureus*; I, intermediate; MRSA, methicillin-resistant *Staphylococcus aureus*; MRCoNS, methicillin-resistant CoNS; MSCoNS, methicillin-sensitive CoNS; MSSA, methicillin-sensitive *S. aureus*; VISA, vancomycin-intermediate *S. aureus*; R, resistant; S, susceptible; –, no interpretive criteria per Clinical and Laboratory Standards Institute.

**TABLE 2:** Summary of the activity of MRS-2541 and comparators against additional *Staphylococcus* spp. isolates^*a*^

Organism	*N*	Drug	MIC (µg/mL)			S/I/R (%)
			Range	MIC_50_	MIC_90_	
*Staphylococcus epidermidis*	15	MRS-2541	0.06 to 0.12	0.06	0.12	–
		Levofloxacin	0.12 to >16	8	>16	33.3/6.7/60.0
		Oxacillin	0.06 to >32	4	16	26.7/–/73.3
		Erythromycin	0.25 to >16	>16	>16	0/26.7/73.3
		Clindamycin	0.12 to >16	2	>16	46.7/6.7/46.7
		Vancomycin	1 to 2	1	2	100/0/0
		Daptomycin	0.5 to 1	1	1	100/–/–
		Linezolid	0.5 to 1	1	1	100/–/0
*Staphylococcus haemolyticus*	10	MRS-2541	0.06 to 0.12	0.12	0.12	–
		Levofloxacin	0.06 to 16	0.12	8	70.0/0.0/30.0
		Oxacillin	0.12 to >32	1	>32	40.0/–/60.0
		Erythromycin	0.12 to >16	>16	>16	10.0/10.0/80.0
		Clindamycin	0.06 to 4	0.06	0.25	90.0/0.0/10.0
		Vancomycin	0.5 to 2	0.5	1	100/0.0/0.0
		Daptomycin	0.25 to 0.5	0.25	0.5	100/–/–
		Linezolid	0.5 to 1	0.5	1	100/–/0.0
*Staphylococcus hominis*	10	MRS-2541	0.06 to 0.25	0.12	0.25	–
		Levofloxacin	0.06 to 8	0.25	8	50.0/10.0/40.0
		Oxacillin	≤0.03 to >32	0.25	>32	60.0/0.0/40.0
		Erythromycin	0.12 to >16	>16	>16	30.0/0.0/70.0
		Clindamycin	0.06 to >16	0.012	>16	60.0/0.0/40.0
		Vancomycin	0.25 to 1	0.5	1	100/0.0/0.0
		Daptomycin	0.25 to 0.5	0.25	0.5	100/–/–
		Linezolid	0.5 to 2	1	2	100/–/0.0

^*a*^ I, intermediate; R, resistant; S, susceptible; –, no interpretive criteria per Clinical and Laboratory Standards Institute.

**TABLE 3:** Summary of activity of MRS-2541 and comparators against *Enterococcus* spp. isolates^*a*^

Organism	*N*	Drug	Phenotype	MIC (µg/mL)			S/I/R (%)
				Range	MIC_50_	MIC_90_	
*E. faecalis*	15	MRS-2541	Overall	≤0.016 to ≤0.016	≤0.016	≤0.016	–
			VSE (*N* = 11)	≤0.016 to ≤0.016	≤0.016	≤0.016	–
			VRE (*N* = 4)	≤0.016 to ≤0.016	≤0.016	–	–
		Levofloxacin	Overall	0.5 to >16	8	>16	40.0/6.7/53.3
		Ampicillin	Overall	1 to 2	1	2	100/–/0.0
		Erythromycin	Overall	0.12 to >16	4	>16	20.0/33.3/46.7
		Clindamycin	Overall	8 to >16	>16	>16	–
		Vancomycin	Overall	0.5 to >32	1	>32	73.3/0.0/26.7
		Daptomycin	Overall	0.5 to 2	1	2	100/0.0/0.0
		Linezolid	Overall	1 to 2	2	2	100/0.0/0.0
*E. faecium*	15	MRS-2541	Overall	≤0.016 to >16	≤0.016	>16	–
			VSE (*N* = 10)	≤0.016 to >16	≤0.016	>16	–
			VRE (*N* = 5)	≤0.016 to ≤0.016	≤0.016	–	–
		Levofloxacin	Overall	0.03 to >16	>16	>16	33.3/0.0/66.7
		Ampicillin	Overall	1 to >32	>32	>32	40.0/–/60.0
		Erythromycin	Overall	1 to >16	>16	>16	0.0/20.0/80.0
		Clindamycin	Overall	0.06 to >16	>16	>16	–
		Vancomycin	Overall	0.5 to >32	0.5	>32	60.0/6.7/33.3
		Daptomycin	Overall	0.12 to 8	2	4	–/93.3*/6.7
		Linezolid	Overall	2 to 8	4	4	33.3/60.0/6.7

^*a*^ I, intermediate; R, resistant; S, susceptible; VRE, vancomycin-resistant *Enterococcus*; VSE, vancomycin-susceptible *Enterococcus*; –, no interpretive criteria; *, susceptible dose dependent per Clinical and Laboratory Standards Institute.

**TABLE 4:** Summary of activity of MRS-2541 and comparators against *Streptococcus* spp. isolates^*a*^

Organism	*N*	Drug	Phenotype	MIC (µg/mL)			S/I/R (%)
				Range	MIC_50_	MIC_90_	
*S. pneumoniae*	30	MRS-2541	Overall	0.06 to >16	0.25	0.5	–
			PSSP (*N* = 12)	0.06 to 0.5	0.25	0.5	–
			PISP (*N* = 5)	0.25 to >16	0.5	–	–
			PRSP/MDRSP (*N* = 13)	0.25 to >16	0.5	0.5	–
		Levofloxacin	Overall	0.5 to 8	1	1	96.7/0.0/3.3
		Penicillin	Overall	≤0.004 to >4	0.25	4	40.0/16.7/43.3
		Erythromycin	Overall	≤0.016 to >16	0.25	>16	50.0/0.0/50.0
		Clindamycin	Overall	≤0.016 to >16	0.06	>16	53.3/0.0/46.7
		Vancomycin	Overall	≤0.03 to 0.5	0.25	0.5	100/–/–
		Daptomycin	Overall	0.06 to 0.25	0.12	0.12	
		Linezolid	Overall	0.12 to 1	0.5	1	100/–/–
Beta-hemolytic streptococci	30	MRS-2541	Overall	0.5 to 8	2	8	–
			ERY-S (*N* = 15)	0.5 to 8	0.5	8	–
			ERY-R (*N* = 13)	0.5 to 8	8	8	–
		Levofloxacin	Overall	0.25 to 2	0.5	1	100/0.0/0.0
		Penicillin	Overall	≤0.004 to 2	0.016	0.03	96.6/–/–
		Erythromycin	Overall	≤0.016 to >16	0.06	>16	50.0/6.7/43.3
		Clindamycin	Overall	0.03 to >16	0.06	>16	76.7/0.0/23.3
		Vancomycin	Overall	0.25 to 2	0.25	0.5	96.6/–/–
		Daptomycin	Overall	0.03 to 1	0.12	0.25	100/–/–
		Linezolid	Overall	0.5 to 1	1	1	100/–/–
Viridans group streptococci	30	MRS-2541	Overall	≤0.016 to >16	0.25	16	–
		Levofloxacin	Overall	0.5 to 16	1	2	93.3/0.0/6.7
		Penicillin	Overall	≤0.004 to 4	0.06	2	63.3/33.3/3.3
		Erythromycin	Overall	≤0.016 to >16	0.03	4	63.3/10.0/26.7
		Clindamycin	Overall	≤0.016 to >16	0.03	16	73.3/0.0/26.7
		Vancomycin	Overall	0.25 to 8	0.5	1	93.3/–/–
		Daptomycin	Overall	0.03 to 2	0.25	2	86.7/–/–
		Linezolid	Overall	0.25 to 2	1	2	100/–/–

^*a*^ I, intermediate; MDRSP, multidrug-resistant *S. pneumoniae*; PISP, penicillin-intermediate *S. pneumoniae*; PRSP, penicillin-resistant *S. pneumoniae*; PSSP, penicillin-susceptible *S. pneumoniae*; R, resistant; S, susceptible; –, no interpretive criteria per CLSI.

**TABLE 5:** Summary of activity of MRS-2541 and comparators against additional *Streptococcus* spp. isolates^*a*^

Organism	*N*	Drug	MIC (µg/mL)			S/I/R (%)
			Range	MIC_50_	MIC_90_	
*S. pyogenes*	15	MRS-2541	0.5 to 2	0.5	0.5	–
		Levofloxacin	0.5 to 2	0.5	1	100/0.0/0.0
		Penicillin	≤0.004 to 0.016	0.008	0.016	100/–/–
		Erythromycin	≤0.016 to >16	0.03	>16	80.0/0.0/20.0
		Clindamycin	0.03 to >16	0.06	>16	80.0/0.0/20.0
		Vancomycin	0.25 to 0.5	0.25	0.5	100/–/–
		Daptomycin	0.03 to 0.25	0.03	0.12	100/–/–
		Linezolid	0.5 to 1	1	1	100/–/–
*S. agalactiae*	15	MRS-2541	8 to 8	8	8	–
		Levofloxacin	0.25 to 1	0.5	1	100/0.0/0.0
		Penicillin	0.016 to 2	0.03	0.03	93.3/–/–
		Erythromycin	0.03 to >16	2	>16	20.0/13.3/66.7
		Clindamycin	0.03 to >16	0.06	>16	73.3/0.0/26.7
		Vancomycin	0.25 to 2	0.5	0.5	93.3/–/–
		Daptomycin	0.12 to 1	0.25	0.25	100/–/–
		Linezolid	0.5 to 1	1	1	100/–/–
*S. mitis*	13	MRS-2541	0.12 to >16	0.5	>16	–
		Levofloxacin	1 to 8	1	1	92.3/0.0/7.7
		Penicillin	0.0008 to 4	0.12	2	61.5/30.8/7.7
		Erythromycin	≤0.016 to >16	0.5	>16	46.2/7.7/46.2
		Clindamycin	≤0.016 to >16	0.03	16	84.6/0.0/15.4
		Vancomycin	0.25 to 0.5	0.5	0.5	100/–/–
		Daptomycin	0.25 to 1	0.25	1	100/–/–
		Linezolid	0.25 to 1	1	1	100/–/–
*S. salivarius*	6	MRS-2541	0.06 to 2	0.12	–	–
		Levofloxacin	0.5 to 2	1	–	100/0.0/0.0
		Penicillin	≤0.004 to 0.25	0.06	–	83.3/16.7/0
		Erythromycin	≤0.016 to 0.03	≤0.016	–	100/0.0/0.0
		Clindamycin	≤0.016 to 0.03	≤0.016	–	100/0.0/0.0
		Vancomycin	0.5 to 1	1	–	100/–/–
		Daptomycin	0.03 to 0.5	0.12	–	100/–/–
		Linezolid	0.5 to 2	2	–	100/–/–
*S. sanguinis*	11	MRS-2541	≤0.016 to 1	0.25	0.5	–
		Levofloxacin	0.5 to 16	1	1	90.9/0.0/9.1
		Penicillin	0.03 to 2	0.12	2	54.5/45.5/0.0
		Erythromycin	≤0.016 to >16	0.25	2	63.6/18.2/18.2
		Clindamycin	≤0.016 to >16	2	>16	45.5/0/54.5
		Vancomycin	0.25 to 8	0.5	8	81.8/–/–
		Daptomycin	0.12 to 8	0.25	8	63.6/–/–
		Linezolid	0.5 to 2	1	2	100/–/–

^*a*^ I, intermediate; R, resistant; S, susceptible; –, no interpretive criteria, per CLSI.

### Staphylococci

As shown in Table 1, MRS-2541 demonstrated an MIC_50/90_ of 0.03/0.06 μg/mL overall against the 45 *S. aureus* isolates tested. This same MIC_50/90_ was observed against the methicillin-susceptible subset (MSSA, *N* = 18), the methicillin-resistant (MRSA; *N* = 27), and vancomycin-intermediate/heterogeneous vancomycin-intermediate (VISA/hVISA; *N* = 12) subsets of isolates. The MRS-2541 MIC values were lower overall than those observed with the seven comparator antibiotics against the 45 *S. aureus* isolates, as well as the MRSA and MSSA subsets. Levofloxacin, oxacillin, erythromycin, clindamycin, vancomycin, daptomycin, and linezolid had the expected activity, with MIC_90_ values of >16, >32, >16, >16, 4, 2, and 2 μg/mL, respectively, against the 45 *S. aureus* isolates. More than 50% of the isolates in this set of *S. aureus* were resistant to levofloxacin; 60% were resistant to oxacillin, and 66.7% were erythromycin resistant.

MRS-2541 had the most potent activity against the 35 coagulase-negative *S. aureus* (CoNS) when examined against the seven comparator compounds, with MIC_50_ and MIC_90_ values of 0.12 μg/mL (Table 1). These same MIC_50/90_ values were observed for MRS-2541 against the 14 methicillin-susceptible CoNS and the 21 methicillin-resistant CoNS. Sixty percent of these isolates were oxacillin resistant; 45.7% were resistant to levofloxacin; and 74.3% were resistant to erythromycin. The comparators oxacillin, levofloxacin, and erythromycin had appropriately high MIC_90_ values of >32, 16, and >16 µg/mL, respectively. Among these isolates, there was no resistance to vancomycin, daptomycin, or linezolid, and these MIC_90_ values were 1 to 2 μg/mL.

Table 2 summarizes the activity of MRS-2541 against isolates of *Staphylococcus epidermidis* (*N* = 15), *Staphylococcus haemolyticus* (*N* = 10), and *Staphylococcus hominis* (*N* = 10). MRS-2541 exhibited MIC_50/90_ values of 0.06/0.12 μg/mL against the *S. epidermidis* tested overall; this compares with MIC_90_ values of 16 to >16 µg/mL for levofloxacin, oxacillin, erythromycin, and clindamycin (resistance rates of 60.0, 73.3, 73.3, and 46.7%, respectively), and 1 to 2 μg/mL for the remaining comparators, against which there was no resistance in this set of isolates. Against the 10 tested *S. haemolyticus* and the 10 tested *S. hominis*, MRS-2541 had MIC_50/90_ values of 0.12/0.12 and 0.12/0.25 μg/mL, respectively. Both sets of isolates had elevated levels of resistance to oxacillin and erythromycin, and these drugs had associated elevated MIC values. Comparators for which there was no resistance among these isolates (vancomycin, daptomycin, or linezolid) showed MIC_90_ values of 0.5 to 2 μg/mL.

### Enterococci

MRS-2541 against 15 *E. faecalis* demonstrated potent activity against these isolates overall, with MIC_50_ and MIC_90_ values of ≤0.016 µg/mL, including against four VRE isolates (Table 3). Resistance to erythromycin in this group was 46.7%; levofloxacin resistance was 53.3%; and vancomycin resistance was 26.7%. These comparators exhibited MIC_90_ values of >16, >16, and >32 µg/mL, respectively. The MIC_50/90_ values for daptomycin and linezolid were 1/2 and 2/2 μg/mL, respectively.

Against 15 *E. faecium* isolates, MRS-2541 had an MIC_50/90_ value of ≤0.016/>16 µg/mL. Against the 5 VRE isolates, the test article displayed MIC values of ≤0.016 µg/mL, whereas against the 10 VSE isolates, the MIC_90_ value for MRS-2541 was >16 µg/mL due to MIC values of >16 µg/mL against 2 of the VSEs tested. Sixty percent of these isolates were ampicillin resistant; 66.7% were resistant to levofloxacin; and 80% were resistant to erythromycin. The comparators ampicillin, levofloxacin, and erythromycin had elevated MIC_90_ values of >32, >16, and >16 µg/mL, respectively. Daptomycin and linezolid had MIC_50/90_ values of 2/4 and 4/4 µg/mL, respectively.

### Streptococci

Despite the fact that some *S. pneumoniae* strains possess the type 2 MetRS enzyme, which is not sensitive to any of MetRS inhibitors, MRS-2541 had MIC_50/90_ values of 0.25/0.5 μg/mL against the 30 *S. pneumoniae* isolates tested, the same value as those observed with vancomycin (0.25/0.5 μg/mL) against this panel (Table 4). This MIC_50/90_ value was the same against the 12 penicillin-susceptible *S. pneumoniae* and similar to that against the 13 multidrug-resistant/penicillin-resistant *S. pneumoniae* (MDR/PRSP, 0.5/0.5 μg/mL). The MIC_50_ for MRS-2541 against the five penicillin-intermediate *S. pneumoniae* (PISP) isolates tested was 0.5 μg/mL. With only low or no levels of resistance in this panel, levofloxacin, vancomycin, daptomycin, and linezolid displayed good activity (MIC_90_ values 0.12 to 1 µg/mL); resistance levels to penicillin, erythromycin, and clindamycin were 43.3% to 50%, resulting in MIC_90_ values of 4 to >16 µg/mL for these drugs.

MRS-2541 had MIC_50/90_ values of 2/8 μg/mL against the 30 beta-hemolytic *Streptococcus* isolates overall. There was a 43.3% resistance to erythromycin among the isolates in this set. Against the 13 erythromycin-resistant isolates in this panel, activity for MRS-2541 was 0.5 to 8 μg/mL with an MIC_90_ of 8 μg/mL. The same MIC_90_ value of 8 μg/mL was observed for this test article against the 15 erythromycin-susceptible isolates in this set. Resistance rates to other drugs were low; MIC_90_ values for levofloxacin, penicillin, vancomycin, daptomycin, and linezolid were 0.03 to 1 μg/mL. If individual beta-hemolytic species are considered, MRS-2541 had strong activity when testing against *S. pyogenes* (MIC_50/90_, 0.5/0.5 µg/mL) and relatively decreased activity against *S. agalactiae* (MIC_50/90_, 8/8 µg/mL) (Table 5).

Against viridans group streptococci, the MIC_50_ and MIC_90_ for MRS-2541 were 0.25 and 16 μg/mL, respectively. About one-quarter of the isolates were resistant to erythromycin or clindamycin; rates of resistance to other agents were low. Levofloxacin, penicillin, vancomycin, daptomycin, and linezolid had MIC_90_ values of 1 to 2 μg/mL, and those for erythromycin and clindamycin were 4 and 16 μg/mL, respectively. Elevated MIC values were observed in *S. mitis* (MIC_50/90_, 0.5/>16 µg/mL) but not in *S. salivarius* (MIC range of 0.06 to 2 µg/mL) or *S. sanguinis* (MIC range of ≤0.016 to 1) (Table 5).

## DISCUSSION

New therapies with novel targets are desperately needed to treat antibiotic-resistant bacteria. A recent study estimated that 4.71 million deaths were associated with bacterial AMR in 2019 (22). This same group in 2024 estimated that 92 million deaths could be averted before 2050 through better treatment of infections and improved access to antibiotics (22). The Centers for Disease Control and Prevention (CDC) estimated that annual costs due to AMR can be upwards of $4.6 billion (23).

Whereas vancomycin is often prescribed for serious gram-positive infections, including those caused by staphylococci, enterococci, and streptococci, resistance rates are increasing for this antibiotic. Both gram-negative and gram-positive infections require new therapy options to close this gap. *Staphylococcus aureus* and enterococci are the second and fifth most common pathogens causing healthcare-associated infections (HAI). VRE infections account for greater than 50,000 HAIs, and approximately 80% of *E. faecium* HAIs are vancomycin resistant (24–26). With *E. faecium* infections exhibiting high prevalence of vancomycin and β-lactam resistance, additional therapeutic options are needed, especially since linezolid resistance has also been observed to be increasing in enterococci (27).

MRS-2541 is currently under preclinical development for gram-positive ABSSSIs and BSIs. This compound was evaluated for *in vitro* activity alongside appropriate comparators against large panels of gram-positive isolates, enriched for isolates with antibiotic resistance phenotypes. Against isolates of clinical relevance of *S. aureus* (including MRSA and VISA) and coagulase-negative *Staphylococcus*, MRS-2541 was the most potent article evaluated. MRS-2541 also demonstrated the most potent MIC_50/90_ values against the panels of *E. faecalis* and *E. faecium* tested, including vancomycin-resistant isolates; there were only two *E. faecium* strains where MRS-2541 was not active (MIC >16 µg/mL). This is the first report of enterococcal strains that were not sensitive to a MetRS inhibitor. *E. faecium* is known to have a multitude of resistance mechanisms, which could include a MetRS enzyme with a modified active site or more likely upregulated efflux pumps, which is often the reason for lack of sensitivity to a new agent (28, 29). Despite the presence of the MetRS type 2 enzyme in some *S. pneumoniae* (19), MRS-2541 showed good activity overall and behaved similarly against PISP and MDR/PRSP but displayed weaker activity against *S. agalactiae and S. mitis*. All 15 strains of *S. agalactiae* had MICs of 8 μg/mL. This could imply sequence modifications of the MetRS enzyme, efflux pumps that recognize MRS-2541, species differences in gene regulation (30), or a combination of factors. Our inspection of all *S. agalactiae* genomes curated in UniProt (www.uniprot.org) does not indicate the presence of a type 2 MetRS enzyme, although the sampling is limited to nine strains. For *S. mitis*, only 4 of 13 strains were insensitive. Further study is needed to understand the mechanism(s) of the resistance, although, as with *S. agalactiae*, there is no evidence of the presence of a type 2 MetRS based on six *S*. *mitis* genomes in Uniprot. It should be noted that all previously reported MetRS inhibitors are significantly less active against streptococci (16- to 64-fold) relative to their potency versus staphylococci (18, 20, 21). The good activity against *S. pyogenes* (MIC_90_ 0.5 µg/mL) is promising for the desired treatment of ABSSSIs, but for BSIs, identifying the causative organism may be important to ensure successful treatment.

In summary, MRS-2541 demonstrated potent activity against a challenge panel of drug-resistant staphylococci, enterococci, and streptococci, including the lowest MIC_50/90_ values for *S. aureus*, CoNS, and enterococci compared to other standard-of-care agents. Potency did not appear to be affected by other resistance phenotypes, including MRSA, VISA, VRE, and PRSP, showing promise for this agent in the context of antibiotic-resistant infections. These data show the promise of MRS-2541 as a novel compound in an environment of increasing resistance rates and fewer therapeutic options for severe gram-positive infections and position this agent well for its intended indications.

## MATERIALS AND METHODS

### Test organisms

Test organisms consisted of reference strains from the American Type Culture Collection (ATCC, Manassas, VA), the CDC (Atlanta, GA), the Network on Antimicrobial Resistance in *Staphylococcus aureus* (NARSA; BEI Resources, managed by ATCC), and independent non-consecutive clinical isolates from the Microbiologics Repository (MMX, Kalamazoo, MI). The CDC, NARSA, and a subset of the MMX isolates were included to select for particular underrepresented susceptibility phenotypes. QC isolates, including *S. aureus* ATCC 29,213, *E. faecalis* ATCC 29,212, and *S. pneumoniae* ATCC 49,619, were included as necessary during testing (31, 32). MIC values were determined in singleton.

### Test media

Cation-adjusted Mueller-Hinton Broth (CAMHB) was used for MIC testing as described by Clinical and Laboratory Standards Institute (CLSI) (31, 32). CAMHB was prepared by supplementing Mueller-Hinton broth (Becton Dickinson) to a final concentration of 20 mg/L Ca^2+^ (Sigma) and 12.5 mg/L Mg^2+^ (EMD Millipore). For the testing of *Streptococcus* spp., this medium was supplemented with 3% laked horse blood (Hemostat).

### Broth microdilution MIC assay

The MIC assay method followed the procedure described by CLSI (31, 32) and employed automated liquid handlers to conduct serial dilutions and liquid transfers. Automated liquid handlers included the Multidrop 384 (Labsystems, Helsinki, Finland), Biomek 3000, and Biomek FX (Beckman Coulter, Fullerton, CA).

The stock solutions for the comparators were prepared using solvents and diluents recommended by CLSI (32). MRS-2541 was supplied by TSRL and dissolved in DMSO for testing. Levofloxacin, oxacillin, ampicillin, penicillin, erythromycin, clindamycin, and vancomycin were supplied by Sigma. Daptomycin and linezolid were purchased from Selleck Chemical (Houston, TX).

Compounds were evaluated with twofold dilutions across an 11-pt scale. The wells of column 12 contained no drug and served as the organism growth control wells for each respective diluent. Each test plate contained the respective media and test agents for broth microdilution evaluation.

A standardized inoculum of each organism was prepared per CLSI methods (31, 32). Colonies were picked from the primary plate, and a suspension was prepared to equal a 0.5 McFarland turbidity standard. The Biomek 3000 delivered diluted standardized inoculum into each well, targeting a final concentration of ~5 × 10^5^ CFU/mL. Plates inoculated with bacteria were incubated for approximately 20 hours at 35°C. The MIC was visually read and recorded as the lowest concentration where bacterial growth was inhibited.

## Supplementary Material

Reviewer comments

## Data Availability

All data are readily available by contacting the corresponding author.

## SUPPLEMENTAL MATERIAL

Supplemental tables spectrum.03615-25-s0001.xlsx Tables S1 to S3.

OPEN PEER REVIEW reviewer-comments.pdf An accounting of the reviewer comments and feedback.

ASM does not own the copyrights to Supplemental Material that may be linked to, or accessed through, an article. The authors have granted ASM a non-exclusive, world-wide license to publish the Supplemental Material files. Please contact the corresponding author directly for reuse.

## References

[1] Blanes Hernández R, Rodríguez Pérez M, Fernández Navarro J, Salavert Lletí M. 2023. Current approach to skin and soft tissue infections. Thinking about continuity of care. Rev Esp Quimioter 36 Suppl 1:37–45. doi:10.37201/req/s01.10.2023 37997870 PMC10793549

[2] Kaye KS, Petty LA, Shorr AF, Zilberberg MD. 2019. Current epidemiology, etiology, and burden of acute skin infections in the united states. Clin Infect Dis 68:S193–S199. doi:10.1093/cid/ciz002 30957165 PMC6452002

[3] Esposito S, Noviello S, De Caro F, Boccia G. 2018. New insights into classification, epidemiology and microbiology of SSTIs, including diabetic foot infections. Infez Med 26:3–14. 29525792

[4] Silverberg B. 2021. A structured approach to skin and soft tissue infections (SSTIs) in an ambulatory setting. Clin Pract 11:65–74. doi:10.3390/clinpract11010011 33535501 PMC7931029

[5] Vella V, Derreumaux D, Aris E, Pellegrini M, Contorni M, Scherbakov M, Bagnoli F. 2024. The incidence of skin and soft tissue infections in the united states and associated healthcare utilization between 2010 and 2020. Open Forum Infect Dis 11:ofae267. doi:10.1093/ofid/ofae267 38835497 PMC11146672

[6] Esposito S, Bassetti M, Concia E, De Simone G, De Rosa FG, Grossi P, Novelli A, Menichetti F, Petrosillo N, Tinelli M, Tumbarello M, Sanguinetti M, Viale P, Venditti M, Viscoli C, on behalf of the Italian Society of Infectious and Tropical Diseases. 2017. Diagnosis and management of skin and soft-tissue infections (SSTI). A literature review and consensus statement: an update. J Chemother 29:197–214. doi:10.1080/1120009X.2017.1311398 28378613

[7] Nandhini P, Kumar P, Mickymaray S, Alothaim AS, Somasundaram J, Rajan M. 2022. Recent developments in methicillin-resistant Staphylococcus aureus (MRSA) treatment: a review. Antibiotics (Basel) 11:606. doi:10.3390/antibiotics11050606 35625250 PMC9137690

[8] Shoaib M, Aqib AI, Muzammil I, Majeed N, Bhutta ZA, Kulyar MF-E-A, Fatima M, Zaheer C-NF, Muneer A, Murtaza M, Kashif M, Shafqat F, Pu W. 2022. MRSA compendium of epidemiology, transmission, pathophysiology, treatment, and prevention within one health framework. Front Microbiol 13:1067284. doi:10.3389/fmicb.2022.1067284 36704547 PMC9871788

[9] Carcione D, Intra J, Andriani L, Campanile F, Gona F, Carletti S, Mancini N, Brigante G, Cattaneo D, Baldelli S, Chisari M, Piccirilli A, Di Bella S, Principe L. 2023. New antimicrobials for gram-positive sustained infections: a comprehensive guide for clinicians. Pharmaceuticals (Basel) 16:1304. doi:10.3390/ph16091304 37765112 PMC10536666

[10] White E. 2023. Enterococcus. Infectious Disease Advisor. Available from: https://www.infectiousdiseaseadvisor.com/ddi/enterococcus

[11] Mazumder SA. 2023. Intravenous-to-oral switch therapy: overview, antibiotics, antidepressants. Medscape. Available from: https://emedicine.medscape.com/article/237521-overview

[12] Cyriac JM, James E. 2014. Switch over from intravenous to oral therapy: a concise overview. J Pharmacol Pharmacother 5:83–87. doi:10.4103/0976-500X.130042 24799810 PMC4008927

[13] Shariati A, Dadashi M, Moghadam MT, van Belkum A, Yaslianifard S, Darban-Sarokhalil D. 2020. Global prevalence and distribution of vancomycin resistant, vancomycin intermediate and heterogeneously vancomycin intermediate Staphylococcus aureus clinical isolates: a systematic review and meta-analysis. Sci Rep 10:12689. doi:10.1038/s41598-020-69058-z 32728110 PMC7391782

[14] Chahine EB, Dougherty JA, Thornby K-A, Guirguis EH. 2022. Antibiotic approvals in the last decade: are we keeping up with resistance? Ann Pharmacother 56:441–462. doi:10.1177/10600280211031390 34259076

[15] Stevens DL, Bisno AL, Chambers HF, Dellinger EP, Goldstein EJC, Gorbach SL, Hirschmann JV, Kaplan SL, Montoya JG, Wade JC. 2014. Practice guidelines for the diagnosis and management of skin and soft tissue infections: 2014 update by the infectious diseases society of America. Clin Infect Dis 59:147–159. doi:10.1093/cid/ciu296 24947530

[16] Nazli A, Tao W, You H, He X, He Y. 2024. Treatment of MRSA Infection: where are we? Curr Med Chem 31:4425–4460. doi:10.2174/0109298673249381231130111352 38310393

[17] Pulido-Cejudo A, Guzmán-Gutierrez M, Jalife-Montaño A, Ortiz-Covarrubias A, Martínez-Ordaz JL, Noyola-Villalobos HF, Hurtado-López LM. 2017. Management of acute bacterial skin and skin structure infections with a focus on patients at high risk of treatment failure. Ther Adv Infect Dis 4:143–161. doi:10.1177/2049936117723228 28959445 PMC5593224

[18] Molasky NMR, Zhang Z, Gillespie JR, Domagala J, Reyna D, Lipka E, Fan E, Buckner FS. 2024. A novel methionyl-tRNA synthetase inhibitor targeting gram-positive bacterial pathogens. Antimicrob Agents Chemother 68:e00745–24. doi:10.1128/aac.00745-24 39470194 PMC11619354

[19] Gentry DR, Ingraham KA, Stanhope MJ, Rittenhouse S, Jarvest RL, O’Hanlon PJ, Brown JR, Holmes DJ. 2003. Variable sensitivity to bacterial methionyl-tRNA synthetase inhibitors reveals subpopulations of Streptococcus pneumoniae with two distinct methionyl-tRNA synthetase genes . Antimicrob Agents Chemother 47:1784–1789. doi:10.1128/AAC.47.6.1784-1789.2003 12760849 PMC155832

[20] Critchley IA, Young CL, Stone KC, Ochsner UA, Guiles J, Tarasow T, Janjic N. 2005. Antibacterial activity of REP8839, a new antibiotic for topical use. Antimicrob Agents Chemother 49:4247–4252. doi:10.1128/AAC.49.10.4247-4252.2005 16189105 PMC1251549

[21] Critchley IA, Green LS, Young CL, Bullard JM, Evans RJ, Price M, Jarvis TC, Guiles JW, Janjic N, Ochsner UA. 2009. Spectrum of activity and mode of action of REP3123, a new antibiotic to treat Clostridium difficile infections. J Antimicrob Chemother 63:954–963. doi:10.1093/jac/dkp041 19258353

[22] Murray CJL, Ikuta KS, Sharara F, Swetschinski L, Robles Aguilar G, Gray A, Han C, Bisignano C, Rao P, Wool E, et al.. 2022. Global burden of bacterial antimicrobial resistance in 2019: a systematic analysis. The Lancet 399:629–655. doi:10.1016/S0140-6736(21)02724-0 PMC8841637 35065702

[23] CDC. 2025. CDC partners estimate healthcare cost of antimicrobial-resistant infections. Antimicrobial Resistance. Available from: https://www.cdc.gov/antimicrobial-resistance/stories/partner-estimates.html

[24] Centers for Disease Control and Prevention (U.S.). 2019. Antibiotic resistance threats in the United States, 2019. Centers for Disease Control and Prevention (U.S.)

[25] Magill SS, O’Leary E, Janelle SJ, Thompson DL, Dumyati G, Nadle J, Wilson LE, Kainer MA, Lynfield R, Greissman S, et al.. 2018. Changes in prevalence of health care-associated infections in U.S. Hospitals. N Engl J Med 379:1732–1744. doi:10.1056/NEJMoa1801550 30380384 PMC7978499

[26] Weiner LM, Webb AK, Limbago B, Dudeck MA, Patel J, Kallen AJ, Edwards JR, Sievert DM. 2016. Antimicrobial-resistant pathogens associated with healthcare-associated infections: summary of data reported to the national healthcare safety network at the centers for disease control and prevention, 2011–2014. Infect Control Hosp Epidemiol 37:1288–1301. doi:10.1017/ice.2016.174 27573805 PMC6857725

[27] Bi R, Qin T, Fan W, Ma P, Gu B. 2018. The emerging problem of linezolid-resistant enterococci. J Glob Antimicrob Resist 13:11–19. doi:10.1016/j.jgar.2017.10.018 29101082

[28] Kristich CJ, Rice LB, Arias CA. 2014. Enterococcal infection—treatment and antibiotic resistance. In Gilmore MS, Clewell DB, Ike Y, Shankar N (ed), Enterococci: from commensals to leading causes of drug resistant infection. Massachusetts Eye and Ear Infirmary, Boston. 24649510

[29] Miller WR, Munita JM, Arias CA. 2014. Mechanisms of antibiotic resistance in enterococci. Expert Rev Anti Infect Ther 12:1221–1236. doi:10.1586/14787210.2014.956092 25199988 PMC4433168

[30] Bryan JD, Liles R, Cvek U, Trutschl M, Shelver D. 2008. Global transcriptional profiling reveals Streptococcus agalactiae genes controlled by the MtaR transcription factor. BMC Genomics 9:607. doi:10.1186/1471-2164-9-607 19087320 PMC2627894

[31] Clinical and Laboratory Standards Institute. 2024. CLSI M07 | Methods for dilution antimicrobial susceptibility tests for bacteria that grow aerobically

[32] Clinical and Laboratory Standards Institute. 2025. CLSI M100 | Performance standards for antimicrobial susceptibility testing

